# The Battle with Germs

**Published:** 1892-07

**Authors:** Julia W. Carpenter

**Affiliations:** Cincinnati


### The Battle with Germs.

BY JULIA W. CARPENTER, M.D., OF CINCINNATI.

Read at the Meeting of the Ohio State Medical Society, May 4, 1892.

Knowledge of the cause of infectious diseases is considered one of the achievements of the present day. Chemistry and the microscope, wielded by indefatigable energy, have brought to light some of the little things that confound the mighty. These microscopic travelers have been traced in their wanderings through the labyrinths of the human body and their manner of warfare studied.

It has been shown that when the micro-organisms enter the body those that are not cast out of the system, are attacked by the white blood corpuscles and other wandering cells called phagocytes. These cells take the parasites into their interior, where they are killed and disintegrated. The destruction of the bacteria in the cells is a *chemical* process. In fatal cases the bacteria poison the cells that engulf them ; or when the infection is overwhelming, there is no battle at all, it is simply a surrender at sight.

Again it has been shown that fresh blood, serum, tissue juice, and saliva, have germicide properties, but not to an unlimited degree. The saliva will destroy the typhoid and cholera bacillus if they are not too numerous. The diphtheria bacillus and pneumo-coccus are not veiled, but lose their virulence.

The destruction of the bacteria by the fluids of the body is also a chemical process. The first, called the phagocytic theory, and the second called the chemical theory do not conflict. The first is a chemical process inside the cell, the other a chemical process outside the cell, but the fluids of the body contain the secretions of cells.

These are the methods of the destruction of the germs in the body, but the reason why the cells win the battle in one case and lose it in another, cannot be demonstrated with our present microscopes and chemical tests. It depends on a yet unknown something, called for convenience vital force, of which some persons have more, some less. The highest degree of vital force which gives resistance to disease is called natural immunity. This is seen when many are exposed to a poison, as small-pox or diphtheria. All do not take the disease, some have the normal vital power that offers the necessary resistance. Living tissue in a healthy state can destroy septic bacteria. If this were not so every one would have tuberculosis, for no one escapes breathing, at some time, air full of the tubercle bacilli. It is when the vitality of the body is lowered that that certain something is wanting that can destroy micro-organisms. The vital force of the cells is the protecting power.

To prevent the inroads of infectious diseases there are only two ways, one pertaining to germs and one to the individuals. As to the germs, they must either be destroyed outside the body or forced to keep at a respectful distance. As to individuals they must cultivate the vital power that gives immunity. Can either of these things be done?

As to the first, are there any ways to destroy germs outside of the body available for every-day life for every one? What say the indefatigable workers in the field of bacteriology? They tell us that one of the most powerful destroyers of bacterial life is—*sunshine*. For instance, speaking of one of the most virulent of germs, the bacillus of anthrax, supposed to live in the soil, they say the anthrax spores are so tenacious of life, so resistant, that nothing in the soil can destroy them, only *sunshine on the surface*.

The little tubercle bacillus, a microscopic line, the dreadful scourge that carries off more than any other one disease, the little rod that calls together the learned from all parts of the earth in great convention, what statements do bacteriologists make about this monarch? The following: "The tubercle bacillus, very tenacious of life, is found alive after being buried long in the earth, or even after exposure to ordinary weather, but is killed after *long exposure to sunlight.*" Are the rooms of consumptives flooded with sunlight for the protection of others, or is the sun shut out to save the carpet? The subject of sunshine as a germicide for every-day life has not been emphasized, but medicinal germicides have been advertised the world over.

Can vital force be cultivated? Without sunshine and fresh air, never. Sunshine, besides being antagonistic to germs, has a wonderful influence on the body, imparting the deficient vital power by a process as unknown as the vital force itself. The human body as well as the vegetable world utilizes sunlight. The whiteness of the plant grown in the cellar, and the green color of the same thing grown in the light, have their counterparts in human beings. Just what is the influence of sunlight on nutrition and nerve force we have no methods at present of estimating, but enough is known to induce physicians to warn people against shutting the sunlight out of their homes for the double reason that it gives vital force to the people and takes it away from germs.

Is the warning needed? Sunlight is shut out of every home as if it were the germs themselves. Take a drive through the suburbs of any city, a sunny winter morning when a little sun, a warm-hearted visitor, should receive a hearty welcome. Every window toward the sun is closed by shutters, shades and curtains, usually all three. If uncovered at all, a square foot is the most. Whether palace or hovel the sun is barred out in all alike. Please notice, if you have not already done so.

Plants with infallible instinct turn to the sun, man with fallible reason turns away from it.

Another forgotten requisite to vital power is fresh air. The best way to prove its necessity is to look at the effects of vitiated air. The carbonic acid from the lungs and the emanations from the body soon make the air unfit to breathe. As it gets to this point it causes languor, headache, an oppressed feeling, and various uneasy sensations. An eminent physiologist very accurately says: " It is a wonderful fact that the body after a time adapts itself to such vitiated air and that one soon can breathe without apparent inconvenience, an atmosphere which, when one first entered it, felt intolerable. Such an adaptation, however, can only take place at the expense of a depression of all the vital functions, which must be injurious if long continued or often repeated." The proof of adaptation is well shown by the experiments of Claude Bernard. A sparrow is placed under a bell glass of such a size that it will live for three hours. If, at the end of the second hour (when it would have lived another hour) it be taken out and a fresh healthy sparrow put in, the fresh healthy sparrow will die instantly. The condition of the sparrow at the end of the first hour, is the state of most persons, and where is the resistance to invading bacteria?

There is a growing dread of air that is most remarkable. People are actually afraid of it. It shows itself on all sides. If a street car is packed, and one, near the door, opens it, it is as quickly closed by another; and until it is actual summer, if one opens a window, all frown and move away, as if it were a leper. Steam cars are no better. Returning in the early spring from a neighboring town, though the temperature was 55° F., each ventilator and window was closed and the stove red hot. As no one was nearer than five seats I ventured to open my window. In five minutes the conductor approached, saying, "I must shut the window, they are complaining of the air." With one window open in the whole car, a draught was not possible. A fish might as well be afraid of water as people of air.

Look at the latest luxury, so-called, in cars, the vestibule train, all closed up, so the whole train is like one long car. In the ordinary car there is now and then a puff of fresh air from the door; now it is a puff from the next car no fresher than your own. What is this atmosphere in which one is shut up for twenty-four hours? Was there ever a car filled with only healthy people? The breath from the healthiest is refuse material, and to this is added the breath from some that have cattarrh, dyspepsia, imperfect teeth, incipient phthisis, and the breath from various other invalids traveling for health. Some smoke is also mixed with this, as the smoking room being small the door is usually open. Opening ones own window cannot destroy this mixture, and inhaling it twenty-four hours puts one's vitality at least as low as that of the sparrow at the end of the first hour, and resistance to the germs inhaled cannot be expected.

People often say they took cold traveling. They had an influenza from the germs they inhaled and could not resist.

What about ventilation at night? The exchange of carbonic acid and oxygen in the lungs is not the same during sleep as when awake. More oxygen is taken in during sleep and stored up for use the next day, but how many give themselves more air at night than in the day time?

A few months ago there was published in one of our journals a statement from some French physician as to a new treatment for phthisis which had brought about wonderfully good results. The new treatment was simply leaving the windows of the sleeping rooms partly open at night. This is amusing in the light of to-day, but as it is the national custom in France to keep all the windows closed at night for fear, as they say, of sore eyes, it is not so strange that he was amazed at the good effect of fresh air at night.

Physicians are by no means without blame in their example as to air. Many ride in close carriages shut up almost the same in sunshine as in storm. In their assembly rooms one might often suppose they were solving the problem as to how long they could breathe the same air over and over and the discussions still continue rational.

There are some physicians, however, in our city, who have the courage to use out-door air as an aid to treatment in some acute diseases, as pneumonia, and have all the windows open. Of course the result is good.

There ought to be a National Department of Health, and one of the chief duties should be to protect people as far as possible from all these so-called pathogenic organisms. And until people have the knowledge to protect themselves they should be protected by law. For instance, garments that have wiped up the unspeakable infectious dust of the streets should not be allowed in any church, theater, school, or any assembly room. Of course persons have the right to stir up such an atmosphere and breathe it if they wish, but they have no right to subject another to it. Dreadful mistakes are made from lack of knowledge. The law of absolute cleanliness holds good outside of surgery. The results of Listerism are needed in more than one department.

Architects should be taught the laws of health so they will not turn beautiful homes into hospitals. Whether they need this instruction or not, look at one house which is only a sample of many. There it stands alone in the center of a large lot, facing the east, so the whole length of the house has the southern exposure so poetically described by novelists. But what is there on this sunny, airy side of the house? All the halls and numerous closets. On the north, where there is no sun, are all the rooms and bay windows. Windows toward the east catch the morning sun, except those on the first floor. No ray of sunshine ever enters there. It is barred out by the porch with a roof so wide and slanting so low that the sun never stoops low enough to peep in. We speak of the coffin-lid crystals of the triple phosphates; the term might well be applied to this kind of a porch roof. If the house could be turned around, the north side on the south, it would be a model.

We have a fine government building occupying half a block. There are large windows, large corridors, but most of the work is done in the interior by gas light.

Architects are not taught that sunlight and fresh air are germicides, and opposed to phthisis, diphtheria, etc., and so they cannot be blamed. In the meantime, however, they go on making the windows smaller and smaller, and the third story of homes almost without the possibility of ventilation.

If the laws of nature cannot be changed, why not follow them? The foundation laws of health are as ignored as if they were yet to be discovered. That sunlight and air are man's best friends, one little dreams. The life of to-day is a cultivated one contrary to nature. It brings about a lowered vitality and susceptibility to the so-called pathogenic micro-organisms, for germs cannot attack actively growing tissues, they cannot affect man unless the tissues have been previously injured.

If the normal vital force gives immunity from these infectious diseases, and its lack gives susceptibility, instead of saying some germs are pathogenic to man, would it not be more correct to say, it is *man* that is so often pathogenic and not the germs.

